# Intraductal papillary mucinous neoplasm of the pancreas (IPMN): clinico-pathological correlations and surgical indications

**DOI:** 10.1186/1477-7819-8-25

**Published:** 2010-04-07

**Authors:** Gian Luca Baiocchi, Nazario Portolani, Guido Missale, Carla Baronchelli, Federico Gheza, Massimiliano Cantù, Luigi Grazioli, Stefano M Giulini

**Affiliations:** 1 Department of Medical and Surgical Sciences, Surgical Clinic, Brescia University, P.le Spedali Civili, 1, 25123 Brescia, Italy; 2 Department of Medical and Surgical Sciences, Endoscopy, Brescia University, P.le Spedali Civili, 1, 25123 Brescia, Italy; 3 Department of Pathology, Brescia Civil Hospital, P.le Spedali Civili, 1, 25123 Brescia, Italy; 4 Department of Radiology, Brescia Civil Hospital, P.le Spedali Civili, 1, 25123 Brescia, Italy

## Abstract

**Background:**

Intraductal papillary mucinous neoplasms (IPMNs) are increasingly recognized entities, whose management remains sometimes controversial, due to the high rate of benign lesions and on the other side to the good survival after resection of malignant ones.

**Methods:**

Retrospective analysis of a prospectively collected Western series of IPMN.

**Results:**

Forty cases of IPMN were analysed (1992-2007). Most patients were symptomatic (72.5%); cholangio-MRI had the best diagnostic accuracy both for the tumour nature (83.3%) and for the presence of malignancy (57.1%). ERCP was done in 8 cases (20%), and the results were poor. Thirteen patients were treated by pancreatic resection and 27 were maintained in follow-up. Total pancreatectomy was performed in 46% of the cases; in situ and invasive carcinoma were recognized in 15.4% and 38.4% of the cases, respectively. The mean follow-up was 42 months (range 12-72). One only patients with nodal metastases died 16 months after the operation for disease progression, while 91.6% of the operated patients are disease free. Out of the 27 not resected patients, 2 out of 4 presenting a lesion at high risk for malignancy died, while the remaining are in good conditions and disease free, with a mean follow-up of 31 months.

**Conclusion:**

Therapeutic indication for IPMNs is mainly based upon radiological evaluation of the risk of malignancy. While the main duct tumours should be resected, preserving whenever possible a portion of the gland, the secondary ducts tumours may be maintained under observation, in absence of radiological elements of suspicion such as size larger than 3 cm, or a wall greater than 3 mm or nodules or papillae in the context of the cyst.

## Background

**Figure 1:**
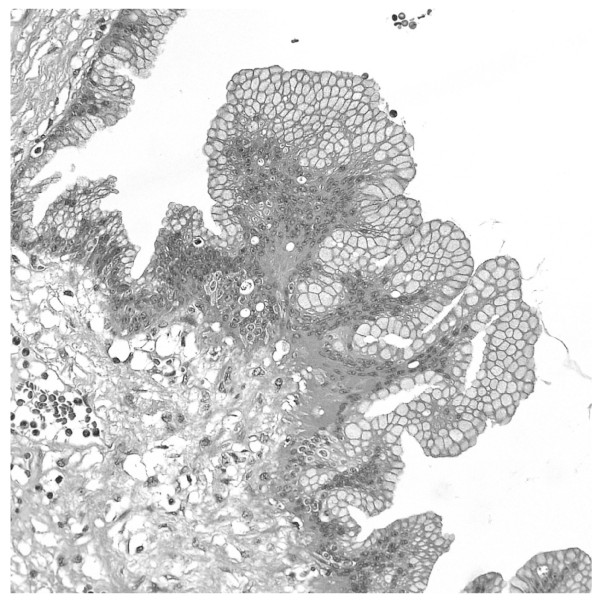
**Main duct IPMN, a Wirsung section shows the epithelial proliferation covered by papillae with abundant mucus production**.

In the group of cystic neoplasms of the pancreas, the intraductal papillary mucinous tumor (IPMN) represents a recently characterized entity; this denomination was introduced in 1996 [1], and comprises a group of lesions that differ from cystic mucinous neoplasms because of a direct communication with the Wirsung duct and the absence of ovarian-type stroma [2]; it is characterized by a papillary growth of the ductal epithelium with rich mucin production and cystic expansion of the interested duct (Fig. 1).

IPMN was firstly described in 1982 [3]; a sharp increase in the frequency of such observations in the following years sets the doubt of a possible new environmental stimulus or a genetic mutation: the hypothesis that before 1980 the IMPNs were simply otherwise classified clashes with the lack of findings of similar tumours in some retrospective revisions [4]. Natural history of this tumour is different from ductal adenocarcinoma: in 90-100% of the cases it is resectable, with survival reaching 80-90% for in situ carcinoma, 50-70% for invasive carcinoma and 40-50% when nodal metastases are already present [5]. Some preoperative indicators of malignancy were proposed, and their accuracy is actually under prospective evaluation [2].

In this paper we present the series of cystic intraductal tumours observed by the Surgical Clinic of the Brescia University, with the aim to underline the clinical problems they set, related both to the therapeutic indication and to the extension of surgical demolition, and to compare the results with those previously reported in Literature.

## Methods

All cases with a definite diagnosis of IPMN (i.e., a cystic neoplasm with a demonstrated communication with or a direct involvement of the Wirsung duct) were taken into consideration. In the period 1992 to 2007, 276 patients affected by pancreas neoplasm have been submitted to surgical exploration, and 186 have been resected, 13 of them with a definitive pathological diagnosis of intraductal papillary mucinous tumor; starting from 2004, on the basis of the literature indications, the cases of IPMN of the secondary ducts of small size have been maintained in follow-up (23 patients); further 4 cases of IPMN suspected for malignancy were not submitted to surgery for an elevated surgical risk (advanced age, cardio-respiratory and liver morbidity); so, the present paper analyzes in total 40 patients with IPMN, 13 treated by surgery and 27 submitted to follow-up.

We considered and recorded as possible risk factors for malignancy smoke, alcoholism, carcinogens exposure, diabetes mellitus, biliary lithiasis, chronic pancreatitis, neoplasms of other organs [5]. CEA and CA 19.9 were recorded in all the patients. Imaging has been analyzed to verify the diagnostic accuracy both for the diagnosis of nature and for the diagnosis of malignancy. In the group of patients submitted to invasive investigations (ERCP with brushing and/or biopsy-aspiration of the cystic content) we considered indications, complications and the influence of the results upon the final therapeutic decision. At the conclusion of the diagnostic workup, IPMNs were subdivided into main duct tumours (MDTs) and branch duct tumours (BDTs). In accordance with the Literature, an high risk for malignancy was hypothesized for all MDT and for BDT larger than 3 cm, or with a wall greater than 3 mm or with nodules of papillae in the context of the cyst [2]. For diagnostic considerations, those criteria were employed also for the cases observed before the publication of the International Consensus Guidelines in 2006; previous cases were retrospectively re-evaluated.

In patients submitted to surgical intervention, the extension of the resection has always been driven by the preoperative evaluation of the dilated Wirsung duct and by the intraoperative histological examination of the pancreatic cut surface in partial pancreatectomy: in case of presence of IPMN in the cut surface, even if without dysplasia, we extended the resection until a completely negative cut section. In the final histopathological examination the WHO classification was followed [6], dividing the cases into 4 groups: IPMAdenoma (dysplasia of low degree), IPMN border-line (dysplasia of moderate degree), in situ IPMCarcinoma and infiltrating IPMCarcinoma. For the 27 patients not submitted to pancreatic resection, the distinction of benign from malignant lesions has been based on the evolution at follow-up, as already proposed [2].

The mean follow-up was 42 months (range 12-72), 47 months (range 15-72) for the operated patients and 31 months (range 12-51) for the observed ones. The follow-up protocol for the operated patients foresees controls at 3, 6, 12, 18, 24 months and subsequently every 12 months up to 5 years. Such controls included clinical examination, tumour markers, abdominal ultrasound or CT or cholangio-MRI, according to the clinical suspicion (in absence of any suspect of recurrence, CT every year for patients with carcinoma and cholangio-MRI every year for patients with benign neoplasm were performed). In the not resected patients, instead, the follow-up was almost entirely founded on the cholangio-MRI, to reduce exposure of the patient to radiations.

## Results

The relative incidence of the IPMNs in the pancreatic surgical series of the Brescia University Surgical Clinic clearly increased from the period 1992-2003 to 2004-2007: 3/122 (2.4%) versus 10/64 resections (15.6%). The 40 patients affected by IPMN were 21 women and 19 males (Table 1). The majority of them were symptomatic (29/40, 72.5%); such percentage was slightly different in resected (61.5%) and not resected patients (77.7%), p = 0.19. The most frequent symptoms were acute pancreatitis (18 cases, 62% of the symptoms and 45% of all the patients with IPMN) and abdominal pain (4 cases, 13.7% of the symptoms). In 11 cases (27.5%) the IPMN was incidentally discovered during ultrasonographic investigation performed for follow-up of other neoplasms (5 cases) or for different reasons (6 cases). An increase of CA 19.9 was documented in 3 patients, while CEA always resulted normal.

**Table 1:** Clinical characteristics of 40 patients with intraductal papillary mucinous neoplasm (IPMN)

	n. pts.	%
*Patients*		
Middle age (range)	68.3 years (36-83)	
Male (mean age)	21 (73 years)	
Female (mean age)	19 (65.9)	
Presence of risk factors		
Biliary lithiasis	7	17.5
Other tumours	5	12.5
Symptomatic at diagnosis	29	72.5
Acute Pancreatitis	18	45.0
Abdominal pain	4	10.0
Diabetes	1	2.5
Diarrhea	1	2.5
Jaundice	1	2.5
Anorexia	1	2.5
Tumor markers		
Raised Ca 19.9	3	7.5
Raised CEA	0	

Table 2 reports data related to the diagnostic course. In most cases the first examination setting the suspicion of IPMN has been the ultrasound (24 cases, 60%), after which CT or MRI were done in all the patients, the diagnostic accuracy of cholangio-MRI being clearly superior (83%), given its ability to show the communication of the cyst with the Wirsung duct. Despite the above examinations substantially brought elements adequate to set the diagnosis of IPMN in 37/40 patients, in many cases the radiologist required a diagnostic confirmation by ERCP. Nevertheless, ERCP was done only in 8 patients (20%), and the results were poor, because a correct spatial definition of the Wirsung duct involvement was obtained only in 4 cases (50%), and the pancreatic brushing was possible only in 5 cases (62.5%), with negative results in all but one (resulting doubtful); in 50% of the cases ERCP was followed by pancreatitis that lasted for various weeks, making it necessary to delay surgical intervention in 2 patients and making more difficult the pancreatic resection in one. In 3 cases we proceeded to aspiration of the cystic content by echo-endoscopy for biochemical and cytological examination, always with negative result. In 7 cases a PET scan was also performed, with 2 positive and 5 negative results [7].

**Table 2:** Diagnostic accuracy of imaging techniques employed in 40 patients with IPMN

	ultrasound (26 pts.)		CT (31 pts.)		MR (24 pts.)	
	n	%	n	%	n	%
Correct diagnosis of IPMN	4	15.4	11	35.4	20	83.3
Correct diagnosis of malignancy°	1/3	33.3	2/4	50.0	4/7	57.1
Correct diagnosis of extension§	1	25.0	9/11	81.8	22	100

° Reference parameter was final histological diagnosis of malignant IPMN

§Reference parameter was macroscopic pathology for resected patients and MRI for the others

At the completion of the pre-operative study, on the basis of the previously reported criteria [2], malignancy could be suspected in 17/40 patients (42.5%) from the morphological characteristics (14 cases), brushing cytology (1 case), CA 19.9 rise (3 cases) and indicative symptoms as jaundice and weight loss (2 cases). Thirteen out of these 17 patients were operated on and 4 were not, because of high surgical risk. Performed interventions are reported in Table 3. In 2 cases the positive intraoperative frozen section examination of the margin imposed to extend the resection up to a total pancreatectomy. A lymphadenectomy was associated to the pancreatic resection in 7 patients, removing the nodes anterior and posterior to the pancreas head, those of celiac artery, hepatic artery, common bile duct and those of the root of the superior mesenteric vein. It was never necessary to associate vascular and visceral resections.

**Table 3:** Surgical and pathological characteristics of 13 patients with resected intraductal papillary mucinous neoplasm (IPMN)

	N.	%
*Therapy*		
Resection	13	32.5
Mean age (range)	68.8 (57-81)	
Total Pancreatectomy	6	46.1
Left Pancreatectomy	5	38.4
Duodeno-pancreatectomy	2	15.4
Lymphadenectomy	7*	53.8
		
*Pathology*		
MDT	10	76.9
BDT	3	23.1
Location		
Head	3	23.1
Body-Tail	5	38.4
Whole Wirsung	5	38.4
WHO classification		
Adenoma	4	30.7
Borderline	2	15.4
In situ carcinoma	2	15.4
Infiltrating carcinoma	5	38.4
Middle diameter (range)	3.92 cm (1.8-6 cm)	
Nodal metastases	1/7	14.3
		
*Postoperative*		
Mortality	1	7.7
Morbidity	4	30.7

*112 nodes were retrieved in total (mean 16/patient)

In all the resected cases the conclusive diagnosis has been IPMN. Ten cases have been classified as main duct tumour (MDT) and 3 as branch duct tumour (BDT). This corresponded to the preoperative classification based on the imaging in all the cases. Location and size are reported in Table 3. If we consider benign the adenoma IPMNs and the border-line IPMNs, and malignant those with carcinoma in situ or infiltrating, 7 resected lesions were malignant (53,8%). Based upon the reported morphological criteria [2], and the final assessment of the radiologist, when clearly expressed, the preoperative diagnostic accuracy in differentiating benign from malignant IPMN was 69.2% (9/13 patients). On the basis of the definitive histological examination, in all the patients the resection has been considered R0. In no case a positive margin was found at the final pathology. One only patient had metastatic nodes.

We complain the death at 62th post-operative day of the patient, already described, submitted to total pancreatectomy for a main duct IPMN, after having experienced acute pancreatitis post ERCP and with a post-operative course complicated by delayed gastric emptying and respiratory sepsis. The overall morbidity of the series (30.7%) includes 2 low-flow fistulas, conservatively treated, a gastrojejunostomy hemorrhage, endoscopically treated, and a pulmonary embolism. Among minor complications, 3 pleural effusions, 1 wound infection and 2 troubles of the glycemic control were recorded. Eleven out of the 12 patients who survived after the resection are alive and disease-free. The patient presenting nodal metastases had a relapse after 12 months and deceased 16 months after the operation. Out of the 27 not resected patients, 2 out of 4 presenting a lesion at high risk for malignancy died, the first 6 months after the diagnosis for disease progression, the second at 16 months for decompensation of a pre-existing Child C liver cirrhosis. The remaining 25 patients, including the 2 remaining whose lesions presented aspects indicative of potential malignancy, are in good conditions and disease free, with a mean follow-up of 31 months.

## Discussion

Malignant IPMN represents a good surgical indication, as Literature survival data report [8-11], and our series confirms. Nevertheless, controversies remain, relative to the diagnostic protocol to be adopted, to the surgical indication in the IPMNs at low risk of malignancy, and finally to the extension of pancreatic resection.

The first problem is the identification of the tumour. IPMNs are symptomatic in most cases, with acute pancreatitis due to duct obstruction from mucus or from the papillary proliferations. In the present series, less than half of the patients had an episode of acute pancreatitis; among these, only 27.7% had a history of biliary stones and 5.5% of alcoholism. Only in 1 case imaging demonstrated a severe pancreatitis. Thus, the typical presentation has been a recurrent acute pancreatitis, without evident cause, of low or moderate severity, but with a log-standing asymptomatic hyperlipasemia. Such set of symptoms must lead to suspect the existence of an intraductal cause, that can be confirmed by further diagnostic steps, such as cholangio-MRI. The remaining half of the patients with IPMN are asymptomatic, and the tumour is discovered incidentally, often during abdominal ultrasound performed for other reasons [12]. Also in these cases the colangio-MRI has elective indication to demonstrate the intraductal nature of the lesion, eventually communicating with the Wirsung duct.

Cholangio-MRI is confirmed from our data to be superior to CT scan in the study of IPMN patients (Table 2). It doesn't emerge from our experience any substantial advantage from ERCP. The only potential added value of such an invasive procedure, the Wirsung duct brushing, finds heavy limits in its extremely low sensibility. In our experience, in half cases the contrast medium wasn't able to depict the whole cystic or polycystic lesion shown by cholangio-MRI, because of the density of its mucous content. Furthermore, ERCP may harbour in these patients an increased risk of complications (e. g. acute pancreatitis), which can heavily interfere with the eventual surgical option. Fifty per cent of the IPMN patients submitted to ERCP developed a iatrogenic pancreatitis in our experience, percentage much higher than ERCP for other diseases. One of these patients had a postoperative course dramatically complicated, concluded by death in 62^th ^day. Surprisingly, this idea was not previously underlined in the Literature. There is no evidence that further invasive diagnostic procedures, such as wirsungscopy, echo-endoscopy with biopsy or fine needle sample and percutaneous biopsy, may warrant a substantial improvement in diagnostic accuracy [13,14]. Starting from 2006, we planned a prospective study on the use of 18-FDG-PET to differentiate benign from malignant lesions [7]. Such technique is able to demonstrate some increased metabolic signal in nodules or solid papillae lying in the context of the cystic wall [15]. The results gathered from our first 28 patients demonstrated an improvement in specificity compared to cholangio-MRI alone (specificity 43% with the MRI, 100% with MRI plus PET), nevertheless such data are in progress and they are not extensively introduced in this paper.

So, the diagnosis of IPMN is usually based upon the imaging (CT/cholangio-MRI) demonstrating a pancreatic cystic mass, involving a dilated main duct, eventually associated to some filling defects (MDT, principal duct), or a normal Wirsung duct communicating with the cyst lesion (BDT, secondary ducts).

**Figure 2:**
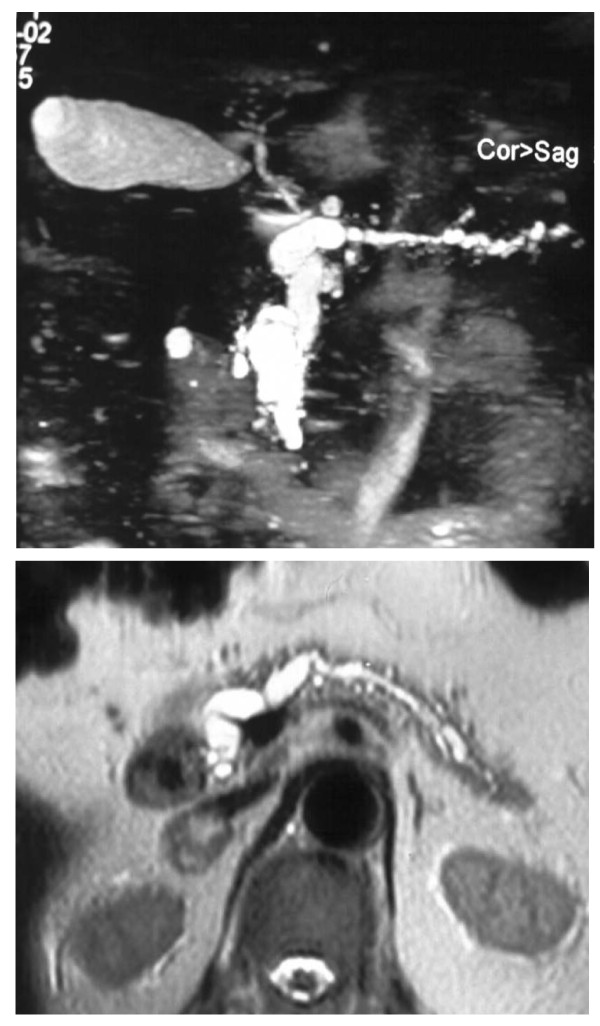
**Colangio-MRI: main duct IPMN involving the whole gland, three-dimensional and axial reconstruction**.

**Figure 3:**
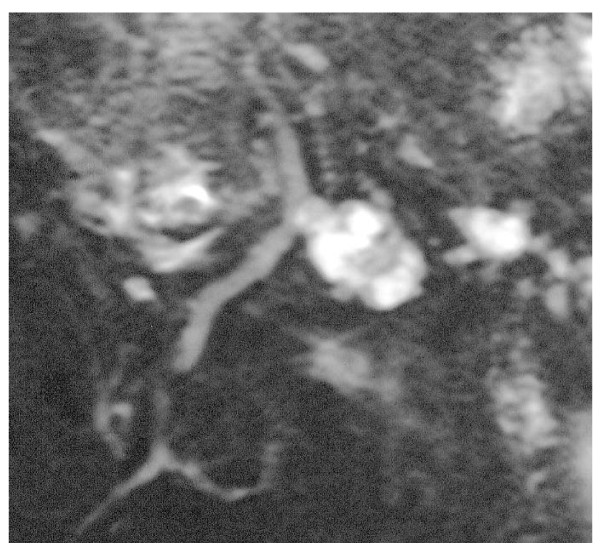
**Colangio-MRI: branch duct IPMN, clear documentation of the communication with the Wirsung duct**.

Many papers analysed the predictive factors for malignancy in IPMN [16-21]. In a recent Japanese retrospective study [22], 17 clinical, radiological and pathological parameters were considered in 64 patients operated on in a 19 years period, defining a prognostic score in which the dimensional cut-off was fixed in 42 mm for the principal cyst and in 6.5 mm for the Wirsung duct, while the CA19.9 discriminant value was 35 U/mls; further parameters considered were jaundice, diabetes, a pathological papilla and the principal duct involvement. Nevertheless, 19/64 patients of this series had a total score of 2 to 4, that didn't allow to define them with certainty as benign or malignant. Among all the analyzed factors, the distinction between MDT (Fig. 2) and BDT (Fig. 3) is confirmed of maximum prognostic value in almost all the series, the second presenting a malignant potential of about 25% (ranging from 6% to 46%), compared to 70% for the former (60-92%) [2]. Not all main duct IPMNs are malignant at diagnosis; nevertheless, resection should be proposed in such cases, considering that: 1) survival of the patients resected for in situ carcinoma is 100%, while it is 60% in presence of infiltrative carcinoma; 2) up to now we cannot obtain by preoperative means a definitive differentiation of dysplasia from carcinoma; 3) the transition from a benign to a malignant form is postulated. The most representative series of main duct IPMN includes 140 patients, of which 60% presented a malignant lesion and 41% nodal metastases [5]. In our series, 10 patients have been submitted to pancreatic resection for main duct tumour: 7 had a carcinoma (2 in situ and 5 infiltrating), multifocal in 3 cases and 1 patient had nodal metastases.

Results are encouraging in this subset of pancreas cancer: just the patient with nodal metastases died 16 months after the intervention, while the remaining 6 are all alive and disease free, with a follow up ranging from 15 and 70 months (mean 42 months). Other studies also reported good outcomes for the resected patients, being 5 year disease free survival rate about 90% for patient with non invasive cancer, 80% for invasive cancers and 65% for patients with nodal metastases [6,9,21]. Thus, with an almost certain diagnosis of intraductal neoplasia involving the Wirsung, a demolitive surgery appears to be justified.

An at least partial conservation of the gland to preserve its exocrine and endocrine function should be part of the strategy of the operation to guarantee a good quality of life to patients who are expected to survive for a long time, and often definitely recover. Nevertheless, the recurrent multifocal involvement of the Wirsung duct sets a limit to the respect of such principle. In our series, we were obliged to extend the resection to the whole gland in 6 out of 10 cases, while in 3 we proceeded to a distal resection and in 1 to an enlarged proximal resection (subtotal duodeno-pancreatectomy). The patients submitted to total pancreatectomy had an obvious whole Wirsung disease from preoperative imaging in 4 cases, while in the remaining 2 the intraoperative frozen sections examination imposed the extension of the pancreas demolition. A recent paper reporting 127 partial pancreatectomies for IPMN shows the difficulty to perform limited resections: in 29% of the cases it was necessary to extend the resection, up to a maximum of 4 re-resections, during the same operation, to obtain a negative margin [23]. In 8% of the this series the Wirsung duct was de-epithelized at the margin and this alteration seems to represent a significant prognostic factor for recurrence; this was reported also previously [24]. In the same series the definitive diagnosis at the margin of the resection was different from the intra-operative results in 6% of the cases. In 19% of 43 patients analyzed by Eguchi through a complex strategy of separate cytology in the different lines of the gland [25], the IPMN resulted to be discontinuous along the Wirsung duct. Moreover the definition of positive section margin is not established: for some Authors it consists in the presence of intraductal papillomatosis even in absence of displasia, for others the displasia of various degree must be present [2].

The branch duct IPMNs (Fig. 3), on the other side, have a better behaviour and prognosis, similar to cystic mucinous neoplasm. In the Verona-Boston series of 145 resected patients, only 22% harboured a malignant cancer, all well characterized in their morphology by a cyst greater than 3 cm, with a wall greater that 3 mm, and nodules or papillae in the cyst [26]. The same Authors reported 163 patient resected for cystic mucinous tumor,17.5% of which were malignant, but never when maximum size was lower than 4 cm and in absence of nodules/papillae [27]. The 26 branch duct IPMNs included in our paper, 3 resected and 23 observed, from the first observation had been classified as benign and they confirmed their benign nature at the histopathology for the resected ones or, if not operated, showing no change at a 28 months mean follow-up. This is in accord with the results of a recent publications from an American multicentric [28] and a Japanese monocentric [29] series; in both studies intraductal branch-duct tumours followed without surgery, clearly showed that it is rarely necessary to perform surgery for a dimensional evolution or for the appearance of radiological elements of suspect (11/70 and 7/82 patients respectively), and that the evolution to carcinoma is rare eventuality (1 carcinoma in situ in both series). Thus, carcinoma in an intraductal branch duct tumour is to be suspected in presence of jaundice and weight loss, or cyst greater than 30 mm or intramural nodules. In patients who doesn't present such characteristics just an instrumental follow-up is indicated.

## Conclusions

The reported series of IPMNs from a Surgical Department confirms the published guidelines: main duct IPMNs have an high risk for malignancy and should be operated on whenever possible, while for branch duct IPMNs clinical and morphological parameters may prove useful to choose the better treatment.

## Competing interests

The authors declare that they have no competing interests.

## Authors' contributions

GLB conceived the study; GLB, NP and SMG participated in the design of the study and drafted the manuscript; FG and MC participated in the patients follow-up; CB carried out the histological analysis; GM carried out the endoscopic examinations; LG controlled all the radiological examinations. All authors read and approved the final manuscript.
